# Supplementary figures and images for: Globins in the marine annelid Platynereis dumerilii shed new light on hemoglobin evolution in bilaterians
Source: BMC Evol Biol. 2020 Dec 29;20:165. doi: 10.1186/s12862-020-01714-4 (PMC7771090; doi:10.1186/s12862-020-01714-4)

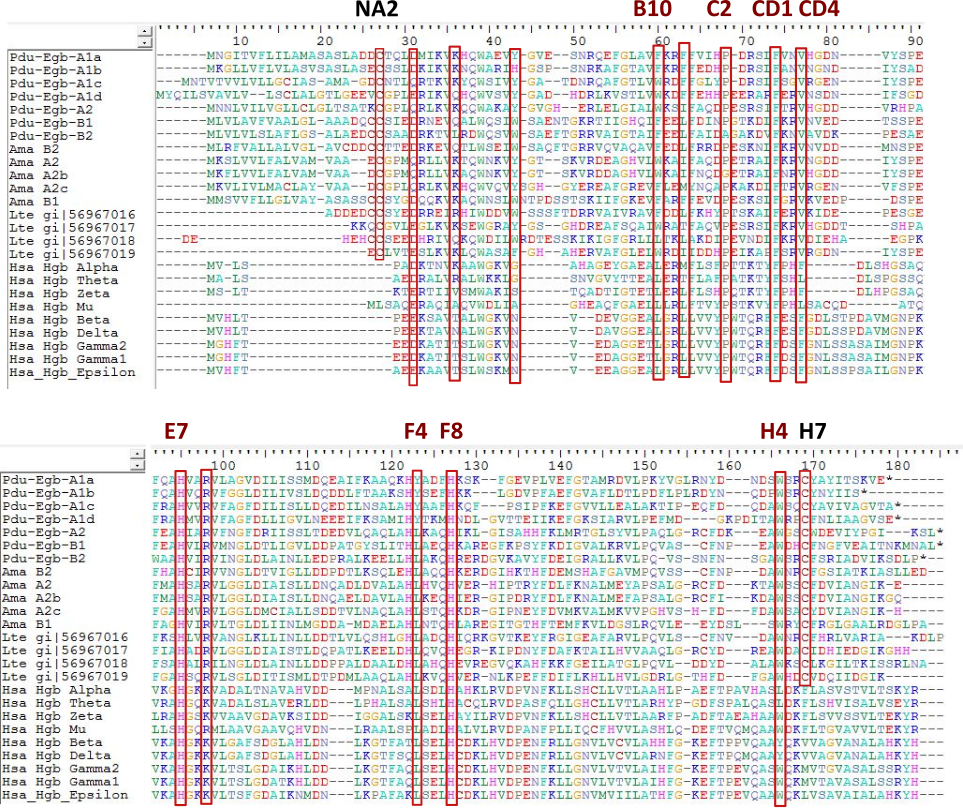

Supplement: Supplementary file 1 — Additional file 1: Amino acids alignment of the Platynereis extracellular globins with the respiratory globins of Arenicola marina, Lumbricus terrestris and human hemoglobins. The highly conserved residues of globins in dark red [78]. The conserved cysteines NA2 and H7 residues known to be involved in the formation of an intrachain disulfide chain in annelids are shown in black [6]. [file 12862_2020_1714_MOESM1_ESM.png]

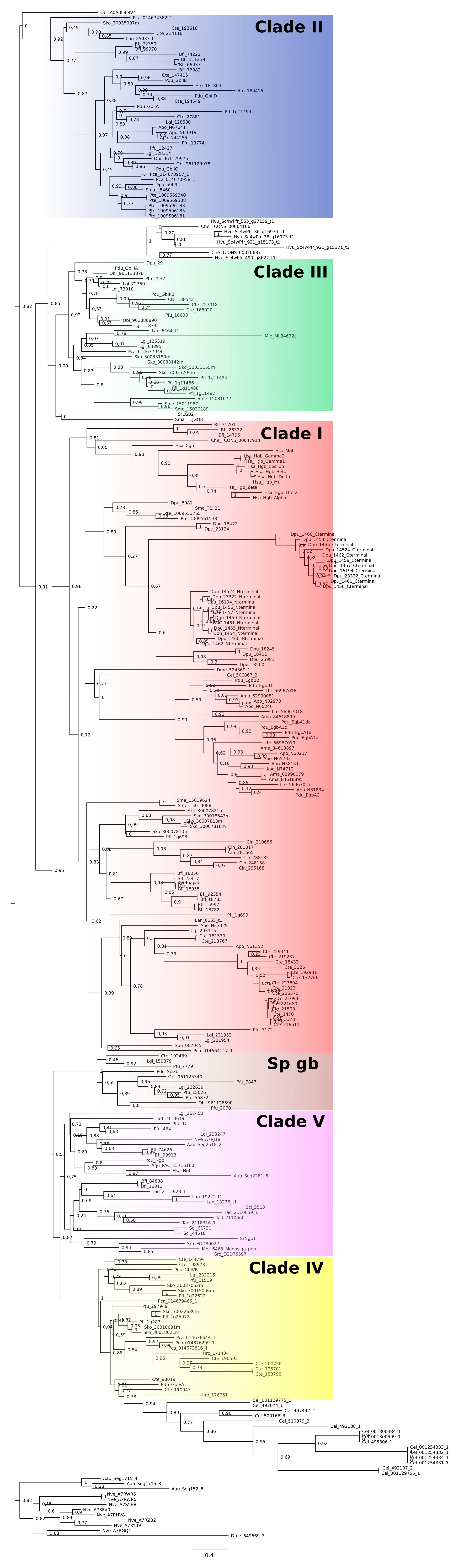

Supplement: Supplementary file 8 — Additional file 8: Maximum likelihood tree using LG model and a SH-like test of a dataset of 293 sequences from 32 metazoan species. The tree is arbitrarily rooted outside of the natural clades. Green and red diamonds indicate a number of nodes that are supported by aLRT values superior to 0.75 and 0.95, respectively. The six clades of globins previously highlighted are again recovered. As hydrozoans (in our analysis, Hydra and Clytia) possess genes related to the clade III. One ctenophore, Mnemiopsis, also display a clade III related gene. One occurrence of a clade I gene in the hydrozoan Clytia appears suspicious as the protein does not show any particular similarity with clade I globins in a blast search. Last, the acoel is shown to possess clade III and V globins. In the absence of a complete genome sequence and the phylogenetic position of acoels being ambiguous, it does not inform on the early evolution of globins. [file 12862_2020_1714_MOESM8_ESM.png]

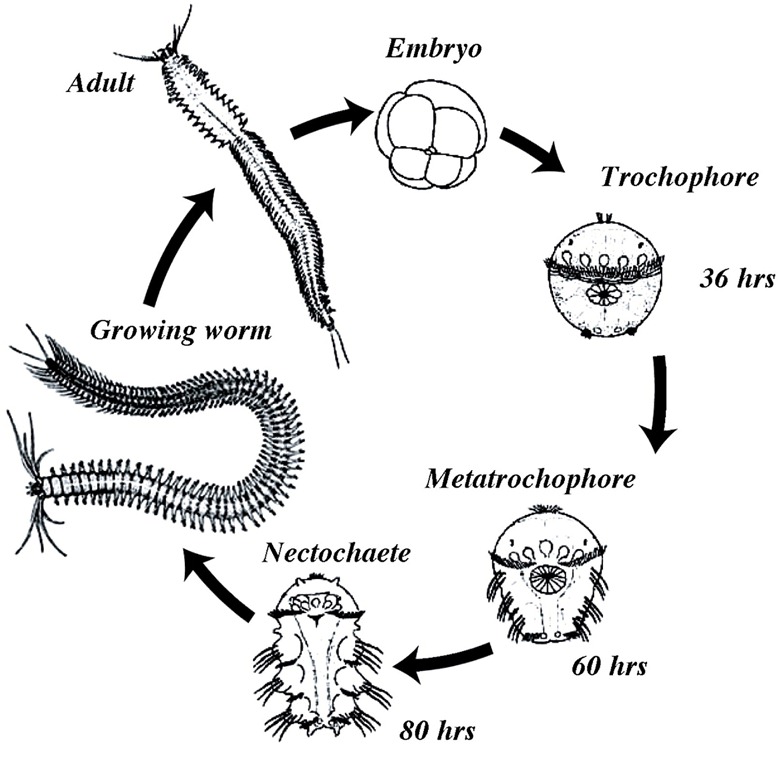

Supplement: Supplementary file 9 — Additional file 9: The life cycle of Platynereis dumerilii. Platynereis dumerilii is a medium-sized annelid that can be easily cultured in the laboratory, giving a large number of offspring all year round. The life cycle is fairly typical of marine annelids. It includes a microscopic (160 μm diameter) lecithotrophic trochophore larva that elongates after 2.5 days in a minute three-segment worm (400 μm long). Once settled in the benthos in a silk tube, the larva will grow by posterior addition of segments and in cross-section to reach a considerably larger size (5–6 cm). The worm lives a relatively sedentary life, feeding on a variety of fresh or decaying food in the benthos for the longest part of its lifespan. Nearing the end of its life, the worm undergoes a rather dramatic sexual metamorphosis: the coelom, from head to tail, entirely fills up with gametes; the worm segmental appendages, called parapodia, change shape and acquire a swimming locomotory function; the gut degenerates as adults do not eat. Both males and females exit their tubes to swarm at the surface of the sea. At this stage they acquire a very fast swimming behaviour and have only a few hours for mating before they die of exhaustion. [file 12862_2020_1714_MOESM9_ESM.jpg]

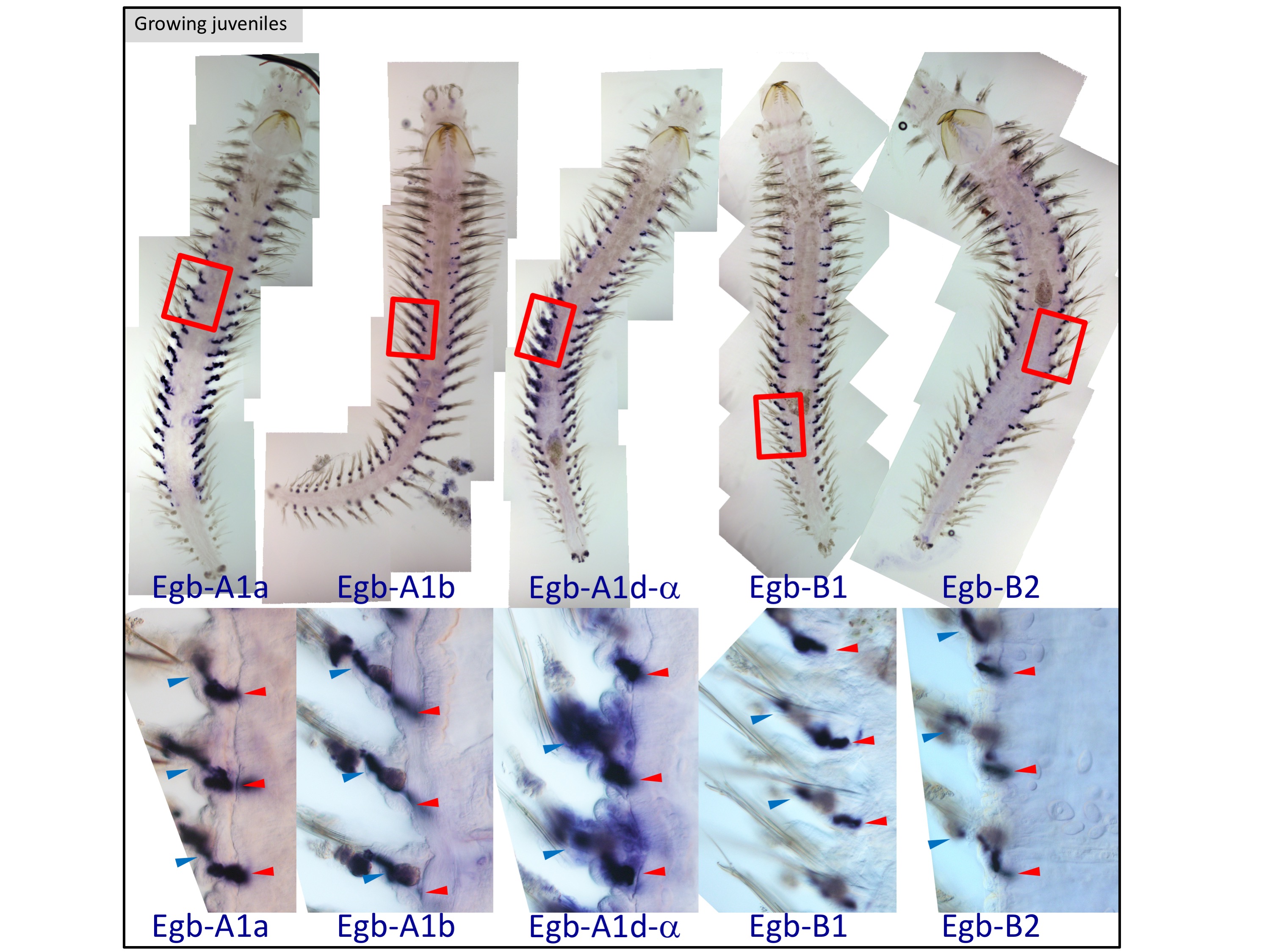

Supplement: Supplementary file 10 — Additional file 10: Expression patterns of five extracellular globin genes in Platynereis dumerilii. WMISH on juvenile stages (30–35 segments). The expression patterns of all extracellular globins are located in the same HPC of transverse trunk vessels (red arrowheads) and parapodial vessels (blue arrowheads). [file 12862_2020_1714_MOESM10_ESM.jpg]
